# A Spray-on, Nanocomposite-Based Sensor Network for *in-Situ* Active Structural Health Monitoring

**DOI:** 10.3390/s19092077

**Published:** 2019-05-04

**Authors:** Wuxiong Cao, Pengyu Zhou, Yaozhong Liao, Xiongbin Yang, Dongyue Pan, Yehai Li, Baojun Pang, Li-min Zhou, Zhongqing Su

**Affiliations:** 1 School of Astronautics, Harbin Institute of Technology, Harbin 150080, China; wuxiong.cao@connect.polyu.hk (W.C.); pangbj@hit.edu.cn (B.P.); 2 Department of Mechanical Engineering, The Hong Kong Polytechnic University, Kowloon, Hong Kong, China; pengyu.zhou@connect.polyu.hk (P.Z.); yaozhong.liao@connect.polyu.hk (Y.L.); xiongbin.yang@connect.polyu.hk (X.Y.); dongyue.pan@connect.polyu.hk (D.P.); yehai.li@connect.polyu.hk (Y.L.); mmlmzhou@polyu.edu.hk (L.Z.); 3 The Hong Kong Polytechnic University Shenzhen Research Institute, Shenzhen 518057, China

**Keywords:** spray-on, nanocomposite ultrasonic sensor, guided ultrasonic waves, structural health monitoring

## Abstract

A new breed of nanocomposite-based spray-on sensor is developed for in-situ active structural health monitoring (SHM). The novel nanocomposite sensor is rigorously designed with graphene as the nanofiller and polyvinylpyrrolidone (PVP) as the matrix, fabricated using a simple spray deposition process. Electrical analysis, as well as morphological characterization of the spray-on sensor, was conducted to investigate percolation characteristic, in which the optimal threshold (~0.91%) of the graphene/PVP sensor was determined. Owing to the uniform and stable conductive network formed by well-dispersed graphene nanosheets in the PVP matrix, the tailor-made spray-on sensor exhibited excellent piezoresistive performance. By virtue of the tunneling effect of the conductive network, the sensor was proven to be capable of perceiving signals of guided ultrasonic waves (GUWs) with ultrahigh frequency up to 500 kHz. Lightweight and flexible, the spray-on nanocomposite sensor demonstrated superior sensitivity, high fidelity, and high signal-to-noise ratio under dynamic strain with ultralow magnitude (of the order of micro-strain) that is comparable with commercial lead zirconate titanate (PZT) wafers. The sensors were further networked to perform damage characterization, and the results indicate significant application potential of the spray-on nanocomposite-based sensor for in-situ active GUW-based SHM.

## 1. Introduction

Defects in engineering structures might not arouse sufficient attention until they deteriorate to a critical and irretrievable level that is prone to cause catastrophic consequences, posing a threat to the structure’s durability and personal safety. Structural health monitoring (SHM) has developed as an emerging approach to pinpoint defects in their embryotic stage without limiting the normal functionality of the structure under inspection. Acousto-ultrasonics-based SHM has come to prominence in the past decades because both acoustic emission (AE) and guided ultrasonic waves (GUWs) exist across a broad frequency regime, enabling monitoring at multi-scale so as to accommodate different demands [1,2,3]. Taking advantage of GUWs, active GUW-based SHM is sensitive to the damage of small dimension, and the damage information can be derived from the GUW signals quantitively, on which basis damage identification and health status perception in different structures can be achieved in-situ with high accuracy.

Targeting quantitative and accurate identification and localization of damage (e.g., bonded mass, hole, delamination, crack) in plates, a number of GUW-based methods have been proposed, such as the delay-and-sum algorithm [4], the probability-based diagnostic imaging (PDI) algorithm [5,6], the time reversal based imaging method [7], and the reconstruction algorithm for probabilistic inspection of defects (RAPID) method [8]. Most of the above mentioned approaches are based on changes in signals, either in a linear domain (e.g., time-of-flight (ToF) [9,10,11]), mode conversion, energy reflection and transmission [12], or in a nonlinear domain (e.g., high order harmonic waves) [13,14,15,16], recording from a spatially distributed sensor network. Because it is easy to extract differential features (e.g., ToFs) from baseline signals and damage modulated signals, the PDI imaging algorithm, an emerging active SHM technique, has been adopted in this paper for damage identification and localization.

As a highly sophisticated technique, acousto-ultrasonics-based SHM is strongly dependent on integrated real-time digital signal processing, pattern recognition and sensor networks. To acquire the GUW signal scattered by damage in an active GUW-based SHM system, development of a sensor network is one of the critical building blocks [17,18,19]. A group of sensors is utilized to build up a sensor network, either embedded internally or attached externally onto the structure, to perceive GUW signals and detect defects in the configured area [8,20,21,22]. With such a sensor network, signals from all sensing paths can be recorded, containing ambient information and system parameters, whereby a perception on the health status of an inspected structure can be developed. There has been an upsurge in efforts to develop sensor networks for active GUW-based SHM, and different kinds of sensors have been utilized, as typified by lead zirconate titanate (PZT) wafers [23], polyvinylidene fluoride (PVDF) piezoelectric films [24], optical fibres [25], piezoelectric transducers [26], carbon nanotube sensors [27,28], and zinc oxide (ZnO) nanocomposite sensors [29], to name a few. To configure an optimized sensor network, two issues should be considered for reaching a balance between “sensing cost” and “sensing effectiveness”: (i) if a sensor network is developed by a dense grid of sensors, not only will extra weight penalty be added to the structure, but the cost of sensors and maintenance also becomes exorbitant; (ii) a sparsely configured sensor network may sometimes “overlook” the damage status of the structure because the information acquired by only a few sensors is inadequate. 

Limited by these facts, a new breed of piezoresistive sensors based on nanocomposites, flexible and small, with low density, high sensitivity, and a broad sensing band, have been developed and fabricated using hot pressing in our previous study [30,31]. Under applied strains, the tunneling effect among nanofiller particles induces a dynamic alteration in the piezoresistive properties of the sensor. Making use of the tunneling effect, particulate movement induced by ultrasonic waves, either of in-plane or out-of-plane mode, can trigger a piezoresistive effect because the nanofillers are randomly and evenly distributed three-dimensionally in the conductive network, which endows the sensor with a capacity to respond to GUW propagating omni-directionally. This type of nanocomposite-inspired sensor has the same sensitivity to the waves from different directions, showing advantage over conventional fiber Bragg grating (FBG)-based sensors to some degree. Application paradigms of our previously developed hot-pressed sensors have highlighted the capability of the nanocomposite-inspired sensor in burgeoning passive AE- or active GUW-based SHM (for both human and engineering assets), tactile sensing, and wearable apparatus, in lieu of conventional sensors [30,32,33,34].

In this study, based on the authors’ previous efforts, a graphene/polyvinylpyrrolidone (PVP) sensor was fabricated by spray coating: a high rate, large area, and cost-effective fabrication approach. In comparison to a hot-pressed sensor, the fabrication process of spray-on sensor is relatively efficient and easy, and the spray-on sensor can be deposited directly on a variety of structures with complex shapes. The morphology and electrical properties of the spray-on graphene/PVP sensor are examined, and the percolation threshold is determined. The spray-on sensor is then validated with commercial PZT wafers in a broadband regime for acquisition of high-frequency GUW signals. On this basis, a dense in-situ monitoring network, utilizing spray-on graphene/PVP sensors, is developed for damage characterization using active GUWs in aluminum alloy plates.

## 2. Spray-on Sensor Fabrication and Characterization

Active SHM features strong penetration and high sensitivity for structural damage, and it is GUWs that contribute to the successful implementation of high precision active SHM. GUWs are of high frequency, yet ultralow magnitude. To perceive GUWs with high accuracy, sensors must be designed and fabricated rigorously. For a nanocomposite-based piezoresistive sensor for the acquisition of dynamic disturbance acquisition, synergy between the nanofiller and the matrix endows the sensor with high sensitivity and enhanced fidelity. PVP is chosen as the matrix in this study to develop the nanocomposite hybrid for the spray-on nanocomposite sensor, as PVP is a kind of water-soluble polymer that can easily form and further stabilize the dispersion of nanocomposites in the solvent without adding extra surfactant because of its amphiphilic groups (hydrophobic methylene group and hydrophilic amide group) [35]. In the authors’ previous study [36,37], a variety of nanofillers such as carbon black (CB), carbon nanotubes (CNTs), and graphene were investigated and proven as promising candidates for developing nanocomposite sensors that can be used for acquiring GUW signals. By virtue of the higher demand for signal quality in active SHM, two-dimensional (2-D) graphene nanoparticles are selected in this study as the nanofiller. The aim is to develop a new breed of sensor with a broad range of responses as well as a high gauge factor, due to two features: (i) unlike CNTs or CB, the 2-D morphology of graphene nanoparticles produces less particle entanglement and aggregation, making the fabrication process more cost-effective and interference-free; (ii) owing to a greater surficial contact area and a higher lateral-thickness aspect ratio, sufficient conductive paths can be engendered under a relatively lower weight ratio, thus achieving a lower percolation threshold with an enhanced electrical conductive performance. 

**Figure 1:**
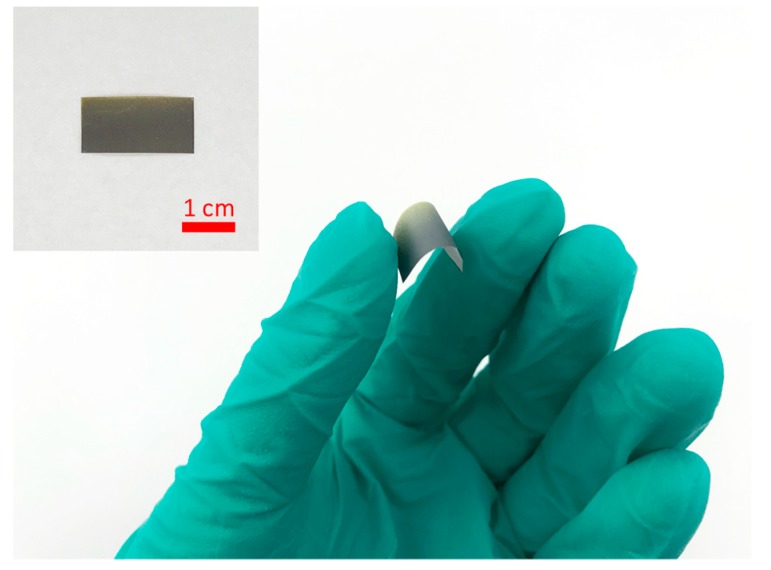
Spray-on graphene/polyvinylpyrrolidone (PVP) sensor on polyimide (PI) film.

The nanocomposite hybrid is prepared by a standard solution mixing process, in which 0.05 g graphene (~1 nm in thickness, 50 mm in diameter, SSA ~ 1200 m^2^/g, purity > 99 wt.%, Suzhou Tanfeng Graphene Technology Co., Ltd., Suzhou, China) is mixed with 0.95 g PVP (PVP K-30, Sigma-Aldrich, St. Louis, MO, USA) in 40 mL ethanol (≥99.8%, Honeywell, Charlotte, NC, USA). The dispersion of graphene and PVP is mechanically stirred for two hours at the rate of 400 rpm, after which the hybrid is sonicated in a sonication bath (Brandson 5800 Ultrasonic Cleaner, 40 kHz, Emerson, St. Louis, MO, USA) for one hour. Polyimide (PI) films with the thickness of 25 μm are chosen as the substrate, due to the good flexibility and chemical stability of PI, making these sensors stable and compatible with different structural surfaces. The prepared hybrid is sprayed directly onto the PI film by an airbrush (HD-130), forming a sensor film with thickness of ~5 μm on the PI substrate. An optical photograph of the spray-on graphene/PVP sensor is presented in Figure 1.

An optimized conductive network formed in a nanocomposite-based piezoresistive sensor effectively enhances the sensor’s sensitivity to a large extent. When the electrical conductive network is at its percolation threshold, the change in tunneling resistance becomes dominant, and thus the sensor, is most sensitive to GUWs. Percolation threshold is a critical volume fraction of insulator-conductor transition corresponding to a small variation of the conductive filler content [38] and at the threshold, the quantum tunneling effect can be triggered under a dynamic disturbance with ultralow magnitude among neighboring non-contacting conductive nanoparticles. The percolation threshold of a nanocomposite follows a power-law relationship with the nanofiller content [39]:

(1) $$\sigma \propto {\left(p-p_{c}\right)}^{t},$$

where σ is the conductivity of the nanocomposite, p the volume fraction of nanofiller, $p_{c}$ the percolation threshold of the composite, and exponential t a constant associated with the dimensionality of the conductive nanocomposites.

**Figure 2:**
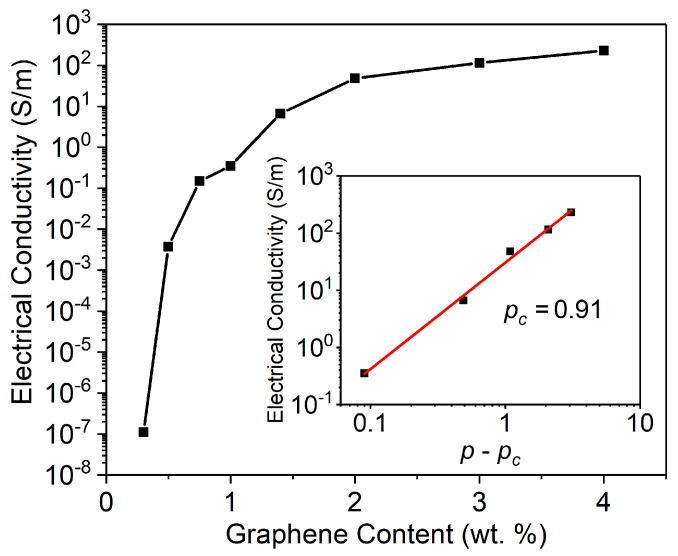
Electrical conductivity of spray-on graphene/PVP sensor with different graphene contents (insert: linear fitting of Figure 2.).

To ascertain the percolation threshold of the spray-on sensor, the spray-on sensors are fabricated with different weight ratios of graphene ranging from 0.3 to 4.0 wt.%. The electrical resistance (R) is measured using a dynamic digital multimeter (Keithley DMM 7510, Tektronix, Beaverton, OR, USA), and contact resistance of the electrodes is negligible, compared with the resistance of the printed sensor, which is of an order of tens of kΩ. The conductivity (σ) is calculated according to σ=l/(R⋅A), where l and A are the distance between two measuring electrodes and the cross-section area. The relationship between σ and the content of graphene is shown in Figure 2. As evident in Figure 2, a remarkable increase in electrical conductivity can be observed when the graphene content is between 0.5 and 1.0 wt.%. With the power-law function linear fitting based on Equation (1), the percolation threshold can be determined as 0.91 wt.%. From the percolation threshold results (0.91 wt.%) determined by percolation theory, a spray-on graphene/PVP sensor with 1.0 wt.% graphene is selected for further investigation.

**Figure 3:**
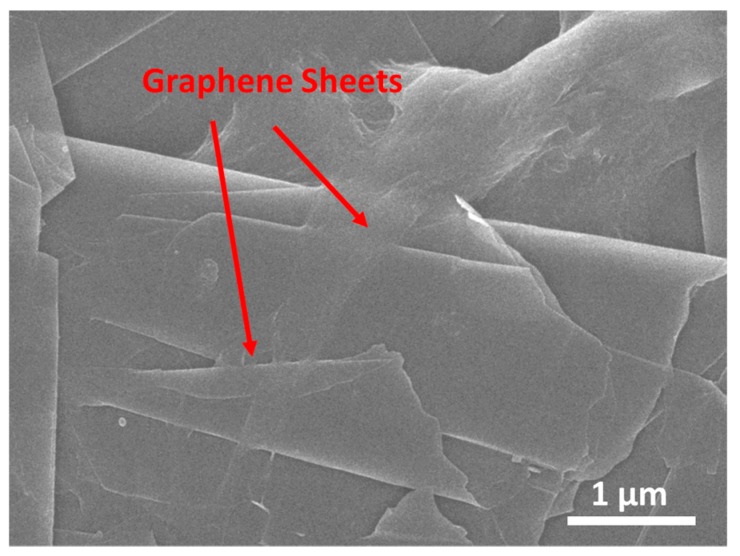
SEM image of the spray-on graphene/PVP sensor with 1.0 wt.% graphene.

The morphology of the spray-on graphene/PVP is characterized using a scanning electron microscope (SEM, JEOL JSM-6490, JEOL, Ltd., Akishima, Japan). Figure 3 presents an SEM image of the spray-on graphene/PVP sensor with 1.0 wt.% graphene. Graphene sheets are sparsely distributed in the PVP matrix, and the 2-D sheets are uniform without entanglement and aggregation, indicating good dispersion of graphene nanofillers in the nanocomposite structure.

## 3. Sensor Calibration for Acquisition of High-Frequency GUW Signals

**Figure 4:**
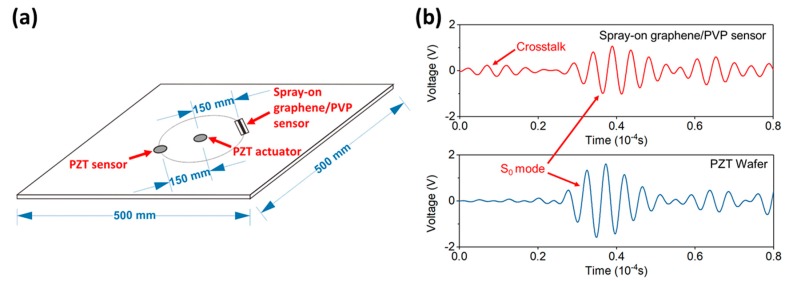
(**a**) Schematic of experimental setup for guided ultrasonic wave (GUW) sensing calibration; (**b**) GUW signals captured by the spray-on graphene/PVP sensor and the PZT wafer.

To achieve damage characterization based on active SHM using the prepared spray-on graphene/PVP sensor, spray-on sensors are calibrated for their in-situ GUW signal sensing performance. Figure 4a shows the experimental setup of the calibration. The spray-on sensor is mounted on the surface of an aluminum plate (500 mm length and width, 1 mm in thickness), together with two PZT wafers (PSN-33, Ø12 mm, 0.48 mm thick, HAIYING Enterprise Group Co., Ltd., Wuxi, China). These two PZT wafers are used as the GUW actuator and sensor, respectively. The distance between the actuator and the sensors is 150 mm. Silver paste is painted onto the graphene/PVP sensor to introduce electrodes and the sensor is connected to a self-developed Wheatstone bridge with 1000 times amplification, as well as an oscilloscope (Agilent^®^ DSO9064A, Agilent Technologies, Santa Clara, CA, USA) for signal acquisition.

During the calibration process, seven-cycle Hanning-windowed sinusoidal tone burst signals are generated by a GUW generation module based on an NI^®^ PXIe-1071 chassis (National Instruments Co., Austin, TX, USA) and further amplified 200 times by a linear power amplifier (Ciprian^®^ US-TXP-3, Ciprian, Grenoble, France). The PZT actuator is connected to the power amplifier and GUWs are excited by the wafer.

**Figure 5:**
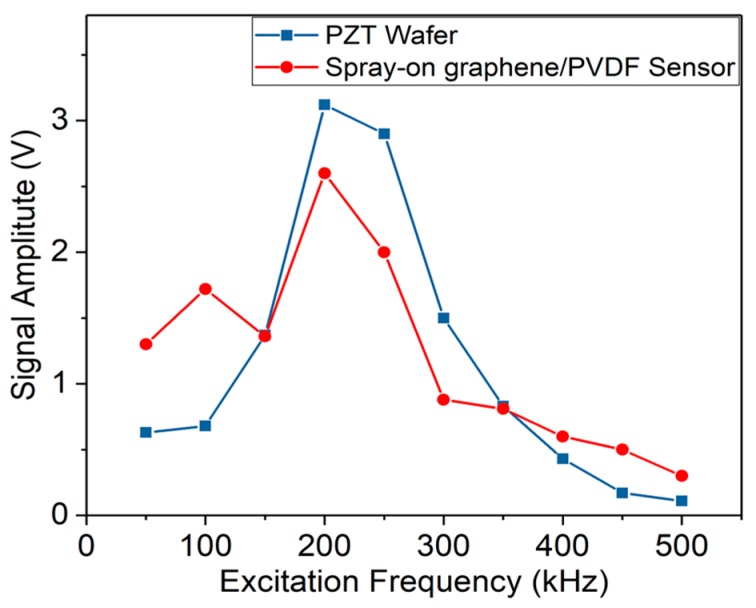
GUW signal amplitude (S_0_ mode) of spray-on graphene/PVP sensor and PZT wafer under excitation frequencies of 50–500 kHz.

Taking the signal acquired at 175 kHz as a typical result, Figure 4b shows the GUW signals captured by the spray-on graphene/PVP sensor and PZT wafer. Crosstalk noise can be observed in the signal acquired by the spray-on PVP/graphene sensor, caused by the signal acquisition system. The first-arriving wave components (denoted by the S_0_ mode, the zeroth-order symmetric Lamb wave mode) captured by these two sensors are very clear and the signal captured by the spray-on PVP/graphene sensor shows almost the same time of arrival (ToA) as the signal from the PZT wafer (with an ignorable discrepancy in the ToA between two signals being 4% only), indicating that the spray-on sensor has good sensitivity and fidelity with no obvious time delay. Figure 5 depicts the GUW signal amplitude of the spray-on sensor and PZT wafer under different excitation frequencies from 50 to 500 kHz. The spray-on graphene/PVP sensor presents a trend of signal amplitude similar to that of the PZT wafer, indicating that this spray-on sensor is capable of GUW perception across a broad sensing band. Note that the slight drop in signal magnitude at 150 kHz can be attributed to the non-uniformity in the nanofiller-formed conductive network during sensor manufacturing, which does not change the holistic consistency in sensing performance between the commercial PZT wafer and the spray-on sensor developed in this study.

## 4. Applications to Damage Characterization

Developed spray-on graphene/PVP nanocomposite film sensors with broad sensing band, serving as GUW receivers, are applied to damage characterization. Considering that this spray-on sensor is lightweight and flexible, a dense sensor network for in-situ acquisition of GUW signals is designed to obtain rich information for characterization of damage with desirable redundancy and hence enhanced reliability of signal acquisition, outperforming conventional piezoelectric transducers in terms of information redundancy and adaptability to complex geometric structure. Using GUW signals captured via the developed sensor network, in conjunction with a ToF-based PDI algorithm, damage can be characterized intuitively and precisely.

### 4.1. Experimental Details

**Figure 6:**
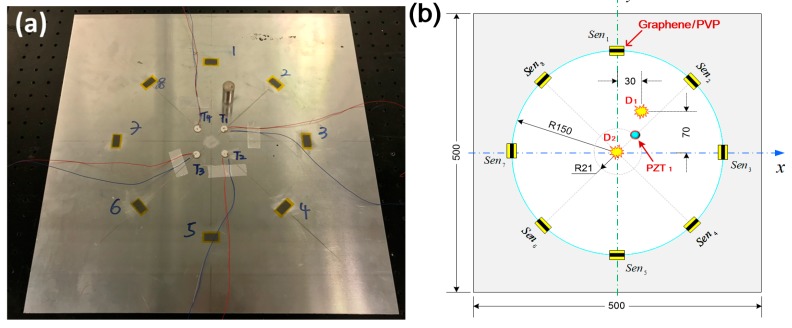
(**a**) 6061T aluminum with the spray-on graphene/PVP sensors and a PZT wafer; (**b**) locations of the actuator, sensors, and damage (unit: mm).

As depicted in Figure 6a, eight graphene/PVP nanocomposite film sensors (denoted by *Sen_i_* (*i* = 1, 2, …, 8)), serving as wave receivers, are surface-mounted on a 6061-T1 aluminum plate (500 × 500 mm in the in-plane dimension, 2 mm in thickness) and one PZT wafer (PSN-33, Ø 12 mm, 0.48 mm thick, denoted by *PZT*_1_) is mounted on the plate as a wave generator, to form a circular sensing network with a total of eight sensing paths. In addition, a mock-up damage (simulated by a bonded mass with radius of 10 mm) is introduced to the plate at a location (30 mm, 70 mm) or (0, 0), denoted by *D*_1_ or *D*_2_, as shown in Figure 6b. Locations of actuator, sensors and damage are displayed in Figure 6b.

**Figure 7:**
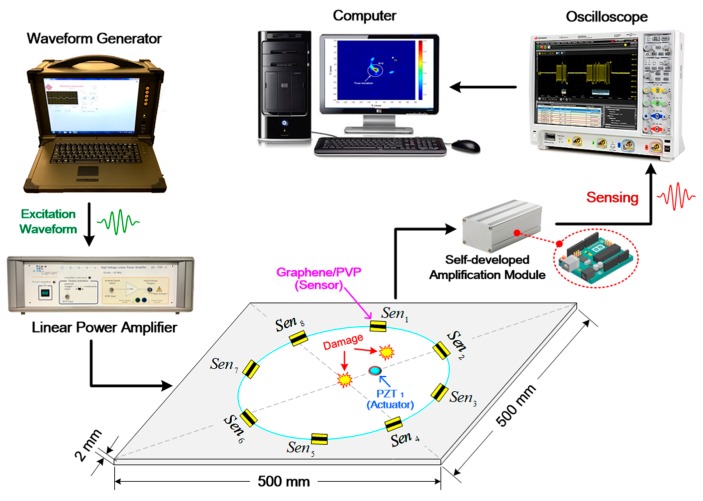
Schematic of the experimental set-up.

A seven-cycle Hanning-window modulated sinusoidal tone burst at a central frequency of 200 kHz is applied to drive the *PZT*_1_ to excite probing GUWs. The selection of 200 kHz lies in the fact that at this frequency, S_0_ mode (the first-arriving wave component) becomes predominant in GUW signals and is of the highest amplitude, ensuring a high signal-to-noise ratio, according to the research outcomes achieved from Section 3. The experimental procedure and configuration of signal acquisition remain the same as that in Section 3, as shown in Figure 7. In the tests, the generated GUW signals are captured via all eight graphene/PVP sensors before and after introducing a mock-up damage to the plate. 

**Figure 8:**
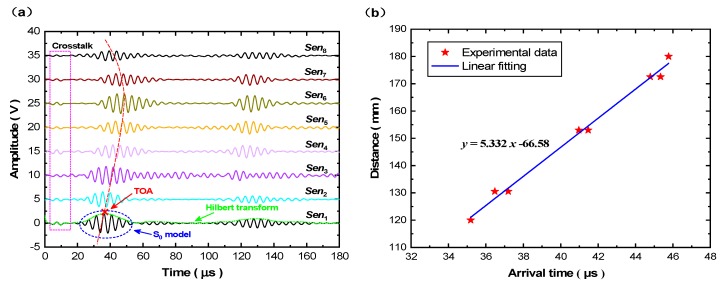
(**a**) GUW signals acquired via all the sensing paths without damage; (**b**) group velocity of S_0_ mode calibrated by linear fitting.

As shown in Figure 8a, GUW signals are acquired via all eight sensing paths before introducing a mock-up damage to the plate, which is considered to be the baselines for the remaining experiments conducted with a damage. The modality of S_0_ mode is clearly observed and becomes predominant in GUW signals, taking as the investigated waves. The propagation velocity of S_0_ mode at 200 kHz is calibrated by linear fitting using ToAs (denoted by symbol “×”, see Figure 8a) of the first wave packet and the distances between the actuator *PZT*_1_ and spray-on sensors, reflected by the distinct slope of the curve, as seen in Figure 8b.

**Figure 9:**
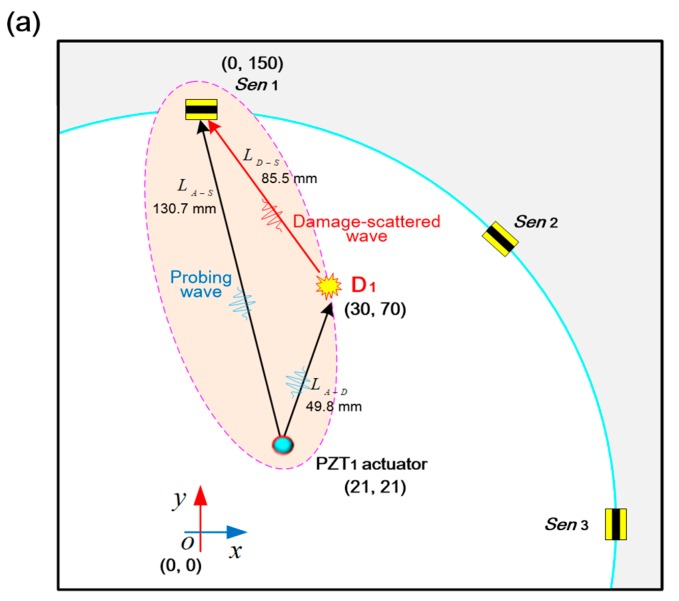

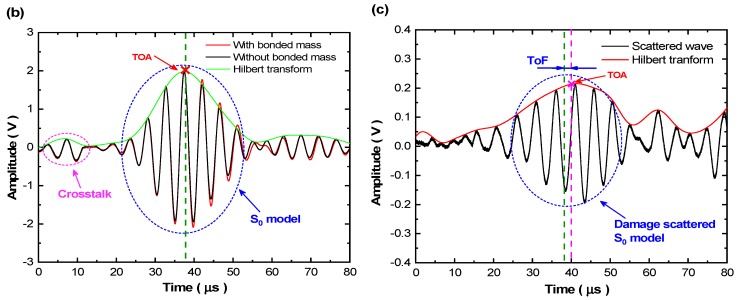
(**a**) Relative position of the actuator *PZT*_1_, sensor *Sen*_1_, and damage *D*_1_ for the sensing path *PZT*_1_-*Sen*_1_; (**b**) representative GUW signals acquired via the path *PZT*_1_-*Sen*_1_ before and after introducing damage; (**c**) scattered wave extracted from these two GUW signals.

A mock-up damage in the plate produces unique wave energy scattering and reflecting phenomena, and forasmuch as the GUW signals acquired by the developed graphene/PVP sensors contains rich information concerning the damage (e.g., location, severity). The experimental procedure and signal processing for two scenarios when the damage is at (30 mm, 70 mm) or (0, 0) respectively remain the same. Therefore, taking the sensing path *PZT*_1_-*Sen*_1_ as a typical example, the relative position of the actuator *PZT*_1_, sensor *Sen*_1_, and damage *D*_1_ is shown in Figure 9a. The probing GUW propagates along the path $L_{A-S}$ directly from the actuator *PZT*_1_ to sensor *Sen*_1_ before introducing damage *D*_1_, and it will also propagate along the path $L_{A-D-S}$ from the actuator *PZT*_1_ to the damage *D*_1_, and then to the sensor *Sen*_1_ after introducing damage *D*_1_. Two GUW signals captured via the sensing path *PZT*_1_-*Sen*_1_ before and after introducing damage *D*_1_ are demonstrated in Figure 9b. Differential feature caused by damage is extracted from these two GUW signals, as shown in Figure 9c. Targeting characterization of damage, ToAs of the damage scattered wave and the directly incipient wave recorded via the same sensing path *PZT*_1_-*Sen*_1_ are used to calculate the ToF, as seen in Figure 9c. A probability-based diagnostic imaging (PDI) algorithm [5,6,40], in conjunction with the ToFs obtained from all sensing paths, is then recalled, whereby the damage is characterized in a grayscale image in terms of the probability of presence.

### 4.2. Probability-Based Diagnostic Imaging

With ToFs extracted from GUW signals obtained via all sensing paths, a triangular equation can thus be established in terms of the relative position of the actuator $A_{i}(x_{A_{i}},y_{A_{i}})$, sensor $S_{i}(x_{S{}_{i}},y_{S{}_{i}})$, and damage $D(x_{D},y_{D})$ as:

(2) $$\left(\frac{L_{A_{i}-D}+L_{D-S_{i}}}{v_{S_{0}}}\right)-\frac{L_{A_{i}-S_{i}}}{v_{S_{0}}}\;=\Delta t_{i},\;(i=1,2,\ldots N),$$

where $L_{A_{i}-D}=\sqrt{{\left(x_{A_{i}}-x_{D}\right)}^{2}+{\left(y_{A_{i}}-y_{D}\right)}^{2}}$, $L_{D-S_{i}}=\sqrt{{\left(x_{D}-x_{S_{i}}\right)}^{2}+{\left(y_{D}-y_{S_{i}}\right)}^{2}}$, and $L_{A_{i}-S_{i}}=\sqrt{{\left(x_{A_{i}}-x_{S_{i}}\right)}^{2}+{\left(y_{A_{i}}-y_{S_{i}}\right)}^{2}}$.

**Figure 10:**
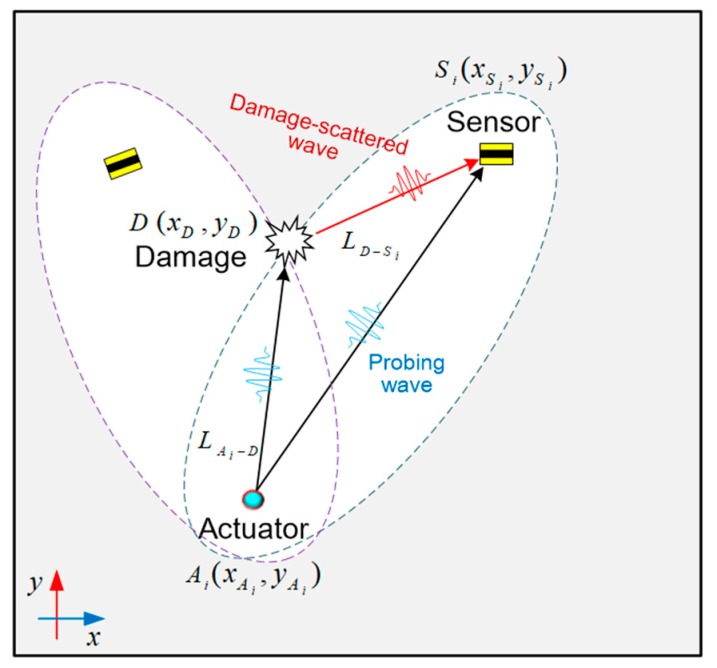
Relative position of the actuator $A_{i}$, sensor $S_{i}$, and damage D for a sensing path.

In the above, $L_{A_{i}-D}$, $L_{D-S_{i}}$ and $L_{A_{i}-S_{i}}$ denote the distances between the actuator $A_{i}(x_{A_{i}},y_{A_{i}})$ and the damage center $D(x_{D},y_{D})$, the damage center and sensor $S_{i}(x_{S{}_{i}},y_{S{}_{i}})$, and the actuator and sensor, respectively. $v_{S_{0}}$ is the group velocity of the incipient S_0_ mode. $\Delta t_{i}$ (i.e., ToF) is to be determined from the GUW signal captured by sensing path $A_{i}-S_{i}$. By solving Equation (2) with the knowledge of $v_{S_{0}}$, $\left(x_{A_{i}},y_{A_{i}}\right)$, and $\left(x_{S{}_{i}},y_{S{}_{i}}\right)$, an elliptical locus with two foci at the actuator $A_{i}$ and sensor $S_{i}$ can be ascertained (see Figure 10), implying all the possible locations of damage in this sensing path. With more elliptical loci from all the sensing paths, the damage location $\left(x_{D},y_{D}\right)$ can be represented by the intersection of ellipses.

In order to visualize the identified damage location in a 2-D grayscale image, a PDI algorithm in introduced, subsequently, the inspected area is virtually and evenly meshed by L×K nodes. The probability of damage occurrence at each mesh node is represented by a field valve, corresponding exclusively to a pixel of a 2-D grayscale image of the inspected area. The nodes that locate on a particular locus have the highest degree of probability (100%) of damage presence from the perspective of the sensing path that produces such a locus; for other nodes, the further the distance to this locus, the less the probability that the sensing path believes there is damage at those nodes. Therefore, the distance ($z_{i}$) from a particular mesh node to the elliptical locus calculated by Equation (2) can be used to quantify the probability of the presence of damage at this node. The field value at each mesh node, which is dependent on the distance ($z_{i}$), can be defined as [6,37]:

(3) $$F(z)=\int_{-\infty }^{z}f(z_{i})dz_{i},$$

where $f(z_{i})=(1/\sigma_{i}\sqrt{2\pi })\exp[-z_{i}^{2}/2\sigma_{i}^{2}]$ depicts the probability density of damage occurrence at mesh node $\left(x_{m},x_{n}\right)$, (m=1,2,…,L; n=1,2,…,K) perceived by the sensing path $A_{i}-S_{i}$, $\sigma_{i}$ the standard variance ($\sigma_{i}=2$ in this paper). In the above, $z_{i}=\sqrt{{\left({\bar{x}}_{i}-x_{m}\right)}^{2}+{\left({\bar{y}}_{i}-y_{n}\right)}^{2}}$ denotes the shortest distance from a spatial mesh node $\left(x_{m},x_{n}\right)$ to the elliptical locus established by this sensing path, where $\left({\bar{x}}_{i},{\bar{y}}_{i}\right)$ is the location on this locus. Therefore, the field value $I(x_{m},y_{m})$ at mesh node $\left(x_{m},x_{n}\right)$ determined by this sensing path is:

(4) $$I(x_{m},y_{m})=1-[F(z_{i})-F(-z_{i})].$$

The image fusion scheme, in accordance with all the sensing paths in Figure 7, is defined as:

(5) $$I{\left(x,y\right)}_{sum}=\sum_{i=1}^{8}I{\left(x,y\right)}_{i},$$

where $I{\left(x,y\right)}_{sum}$ denotes the field value at pixel (x,y) in the ultimate grayscale image. With image fusion, the damage location can be highlighted at image pixels intuitively and precisely.

### 4.3. Results and Discussion

**Figure 11:**
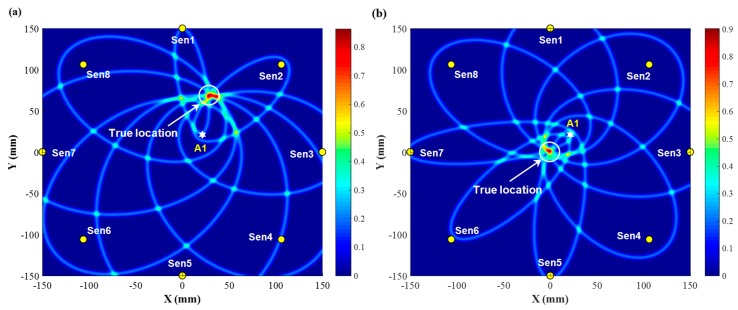
Diagnostic image obtained using the ToF-based PDI algorithm: (**a**) damage at (30 mm, 70 mm); (**b**) damage at (0, 0).

The imaging results of damage constructed by all eight sensing paths of the developed sensor network, using the PDI algorithm based on ToFs, are now presented and discussed. Figure 11 illustrates the identification results for two scenarios when the mock-up damage is at the locations (30 mm, 70 mm) and (0, 0), respectively, showing good agreement with the true locations. Note that for a specific point, the higher the degree of probability of the presence of damage is, the remarkably higher field value with an outstanding pixel is displayed in the diagnostic image, giving users an intuitive and precise perception of the damage location.

**Figure 12:**
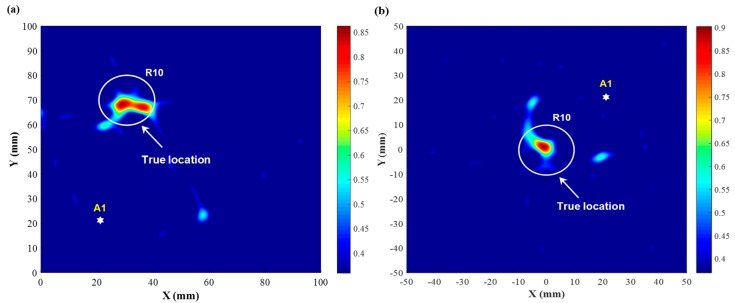
Diagnostic image obtained using the ToF-based PDI algorithm with a threshold value: (**a**) damage at (30 mm, 70 mm); (**b**) damage at (0, 0).

Further setting a threshold value on the diagnostic images in Figure 11, the ultimate resultant probability image is exhibited in Figure 12, where the location and size of the mock-up damage are revealed clearly. Although not predicting the damage size and shape precisely, the imaging results are still able to identify the location of the mock-up damage existing in the inspected region, demonstrating the capacity of the developed nanocomposite sensors for active GUW-based damage localization.

## 5. Conclusions

In this paper, a new type of nanocomposite based piezoresistive sensor for dynamic strain was developed using graphene and PVP and fabricated by a simple spray coating process. Nanocomposite hybrid for the graphene/PVP sensor can be deposited directly onto various surfaces with different shapes with a high rate, demonstrating that the sensor is not only conformable with complex structures, but also can be fabricated in a large area. The quantum tunneling effect in the conductive network endows the spray-on graphene/PVP sensor with the capability of perceiving high-frequency GUW signals up to 500 kHz with excellent sensitivity and accuracy that are comparable with commercial PZT wafers.

The spray-on sensors are then densely networked to detect and localize damage. The experimental results demonstrate that the lightweight and flexible spray-on sensor network can acquire rich information scattered by the damage, and can further indicate accurate locations of damage. Compared to our previously developed hot-pressed sensors, this spray-on sensor features the merits of high processing efficiency, enhanced flexibility, and stability. By virtue of its lightweight, flexible, low fabrication cost (remarkably lower than that when the same number of PZT wafers are used to configure a sensor network), and superior conformability, large quantities of sensors can be directly deposited to form a dense monitoring network to accommodate diverse needs such as ultrasonics-based health monitoring (for both human and engineering assets), tactile sensing, and wearable apparatus, highlighting the effectiveness of the spray-on graphene/PVP sensor for in-situ acousto-ultrasonics-based SHM.
